# Pre-expanded muscle-sparing latissimus dorsi flap in defect reconstruction and its application strategy

**DOI:** 10.1093/burnst/tkae014

**Published:** 2024-06-19

**Authors:** Yashan Gao, En Yang, Shenying Luo, Xin Huang, Yi Min Khoong, Shuchen Gu, Yunhan Liu, Wenzheng Xia, Haizhou Li, Tao Zan

**Affiliations:** Department of Plastic and Reconstructive Surgery, Shanghai Ninth People’s Hospital, Shanghai JiaoTong University School of Medicine, Zhizaoju Road No. 639, Shanghai 200011, China; Department of Plastic and Reconstructive Surgery, Shanghai Ninth People’s Hospital, Shanghai JiaoTong University School of Medicine, Zhizaoju Road No. 639, Shanghai 200011, China; Department of Plastic and Reconstructive Surgery, Shanghai Ninth People’s Hospital, Shanghai JiaoTong University School of Medicine, Zhizaoju Road No. 639, Shanghai 200011, China; Department of Plastic and Reconstructive Surgery, Shanghai Ninth People’s Hospital, Shanghai JiaoTong University School of Medicine, Zhizaoju Road No. 639, Shanghai 200011, China; Department of Plastic and Reconstructive Surgery, Shanghai Ninth People’s Hospital, Shanghai JiaoTong University School of Medicine, Zhizaoju Road No. 639, Shanghai 200011, China; Department of Plastic and Reconstructive Surgery, Shanghai Ninth People’s Hospital, Shanghai JiaoTong University School of Medicine, Zhizaoju Road No. 639, Shanghai 200011, China; Department of Plastic and Reconstructive Surgery, Shanghai Ninth People’s Hospital, Shanghai JiaoTong University School of Medicine, Zhizaoju Road No. 639, Shanghai 200011, China; Department of Plastic and Reconstructive Surgery, Shanghai Ninth People’s Hospital, Shanghai JiaoTong University School of Medicine, Zhizaoju Road No. 639, Shanghai 200011, China; Department of Plastic and Reconstructive Surgery, Shanghai Ninth People’s Hospital, Shanghai JiaoTong University School of Medicine, Zhizaoju Road No. 639, Shanghai 200011, China; Department of Plastic and Reconstructive Surgery, Shanghai Ninth People’s Hospital, Shanghai JiaoTong University School of Medicine, Zhizaoju Road No. 639, Shanghai 200011, China

To the editor

In 2003, Schwabegger *et al*. proposed the muscle-sparing latissimus dorsi (MS-LD) flap [1], which preserved a portion of the latissimus dorsi (LD) muscle around the point where the thoracodorsal artery (TDA) perforator penetrates the muscle, having the advantages of a sufficient flap blood supply and reduced donor-site morbidity. However, the traditional MS-LD flap is still too bulky for the reconstruction of defects of the face and neck, and the size of the traditional flap is sometimes not enough to repair large defects. Thus, combining soft tissue expansion with a vascular supercharging technique, we propose a novel design of MS-LD flap, as well as its application strategy.

In the first stage, an 8 cm incision was made 2 cm anterior of the front edge of the LD muscle, deep to the surface of the serratus anterior muscle, and then a dissection was performed between the serratus anterior and the LD muscle. After detecting the lateral branch of the TDA (usually ~2–3 cm behind the front edge), the LD muscle was cut off, and then a dissection was performed on the superficial surface of the LD muscle. This results in an MS-LD flap consisting of two parts: the ‘musculocutaneous part’ which contains the LD muscle and lateral branch of the TDA, and the ‘cutaneous part’ which does not contain the LD muscle. The expansion stage lasted between 6 and 13 months until the area of the pre-expanded skin was estimated to exceeded 120% of the defect area to ensure adequate coverage.

In the second stage, an incision was made along the incision made in the first stage, and the flap was designed according to the defect size. Following removal of the expander, the lateral branch of the TDA was dissected to a sufficient length, while the medial branch of the TDA was ligated. If necessary, the circumflex scapular artery could be further ligated to get a longer flap pedicle.

Also, the medial and lateral branches of the thoracodorsal nerve were dissected, and the lateral branch of the thoracodorsal nerve was ligated, leaving the medial branch to innervate the preserved LD muscle.

According to needs, the MS-LD flap could be flexibly designed as a free flap or a pedicled flap. For an extremely large flap, when the flap area exceeds the perfusion area (clinical territory) of the lateral branch of the TDA, the lateral intercostal artery perforator (LICAP) could be dissected to harvest a supercharging flap.

Based on the above, we propose classification of pre-expanded MS-LD flap into three types Figure 1a. Type I: a free pre-expanded MS-LD flap with the lateral branch of the TDA as the pedicle. Type II: a pedicled pre-expanded MS-LD flap with the lateral branch of the TDA as the pedicle. Type III: a pre-expanded LICAP supercharged MS-LD flap with the lateral branch of the TDA as the pedicle, which could be designed as either a free flap or a pedicled flap.

**Fig.1:**
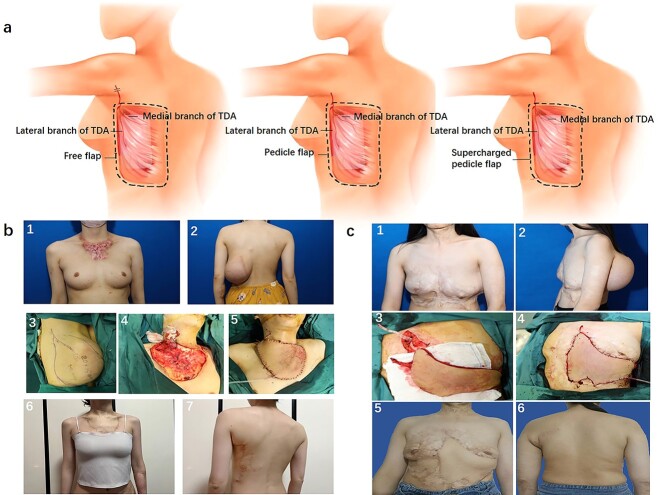
Schematic diagram of flap designs and case reports. (**a**) Schematic diagram of the three muscle-sparing latissimus dorsi (MS-LD) flap designs (from left to right): free MS-LD flap, pedicled MS-LD flap and lateral intercostal artery perforator (LICAP) supercharging MS-LD flap. (**b**) In case 1, a free modified pre-expanded MS-LD flap was used to repair the post-burn scar on the chest wall. (1) Preoperative photo showing scarring on the chest wall scar. (2) The appearance of the donor site following 8 months of expansion. (3) The size of the expanded flap was measured to be 20 × 15 cm. (4) The lesion was excised completely during stage II surgery. (5) The flap was transferred to the chest, while the TDA was anastomosis with the superior thyroid artery. (6) At the 36-month follow-up, the color and texture of the flap resembled those of the surrounding tissue. (7) The donor site showed no obvious scarring at the 36-month follow-up. (**c**) In case 2, a pedicled pre-expanded LICAP supercharged MS-LD flap was used to reconstruct post-burn scars on the chest and abdominal wall. (1) Preoperative photo showing scarring on the chest and abdominal wall. (2) The appearance of the donor area following 6 months of expansion. (3) The lesion was completely excised during stage II surgery. (4) Transfer of the flap to the chest and abdominal wall during stage II surgery, while the LICAP was anastomosis with the second internal mammary artery perforator. (5) At 21-months follow-up, the flap’s color and texture resembled those of the surrounding tissue. (6) The donor site showed no obvious scarring at the 21-month follow-up

Enlargement and thinning of the flap make it suitable for reconstructing large defects with optimal functional and aesthetic outcomes. Skin and soft-tissue expansion in the first stage could help to harvest a large, thin flap and to reduce donor-site morbidity. In our study, the flap could reach a maximum size of 35 × 20 cm, while the donor site could be closed primarily. In addition, the thickness of the flap was significantly reduced after skin expansion, which contributed to improved reconstructive outcomes both functionally and aesthetically.

According to the study by Schaverien *et al*. [2], at least one perforator was found originating from the lateral branch, while 70% of all perforators originated from the lateral branch and 30% from the medial branch. The perfusion range of the perforators of the lateral branch of the TDA was midline, mid-axillary line, C7 level superiorly and level with the iliac crest inferiorly [2]. Also, Ha *et al*. described how a 35 × 19 cm^2^ TDA perforator flap could be harvested safely [3]. In our study, a 4–5 cm width of LD muscle around the lateral branch of the TDA was preserved in the first stage, and then expanded as the ‘musculocutaneous part’, which helps to ensure sufficient flap blood supply. In the second stage, as the medial branch of the thoracodorsal nerve and the vast majority of the LD muscle is preserved, >95% of the LD muscle function could be preserved following flap harvest. Thus, none of the 13 patients using the modified pre-expanded MS-LD flap in our study reported complications at the donor or recipient site (including complications related to tissue expansion, flap necrosis, donor site morbidity or weakness of the LD muscle).

Based on the concept of ‘angiosome of the body’ proposed by Taylor *et al*. [4] and the concept of ‘perforasome theory’ proposed by Schaverien *et al*. [2], the extended MS-LD flaps (type III), which exceed the clinical territory of the lateral branch of the TDA, could be designed as supercharging flaps that connect with the next anatomical territory of the LICAPs, posterior intercostal artery perforators, etc. The flexible selection of the supercharging vessel was based on the characteristics of the defects and the location of the recipient vessel. Our clinical and animal study has also shown that vascular supercharging could augment the blood supply of the perforator flap and also reduce venous compromise [5].

Depending on the the location and size of the defect, different pre-expanded MS-LD flap applications could be selected**.** As mentioned above, the type I free flap, is used primarily to repair defects located far from the back, including limbs, neck and face (Figure 1b). For the reconstruction of defects of the chest and shoulder, we suggest designing a type II pedicled flap due to its proximity to the latissimus dorsi region, avoiding the difficulties and risks of microsurgery. For larger defects that might exceed the perfusion size of the MD-LD flap, we integrate the vascular supercharging technique into the MS-LD flap (type III), which could further ensure the blood supply of the supersized MS-LD flap (Figure 1c). Using the above application strategy, we have successfully used the pre-expanded MS-LD flap to reconstruct various defects.

In conclusion, the modified pre-expanded MS-LD flap could improve functional and aesthetic reconstructive outcomes, with minor recipient and donor site complications, and could be designed flexibly.

## Funding

This study was supported by National Natural Science Foundation of China (82072177, 82272264) and the ‘Hengjie’ Program of Shanghai Health Youth Talent Reward Foundation.

## Authors’ contributions

YG and EY designed the study and were the primary authors of the manuscript. TZ and HL were the chief surgeons and are the corresponding authors. All authors contributed to the enrolment of patients and sample collection. All authors critically reviewed and approved the final manuscript.

## Ethics approval and consent to participate

This study was approved by the Medical Ethics Committee of Shanghai Jiao Tong University School of Medicine, China (SH9H-2018-T47–3). Verbal informed consent was obtained from all patients included in case reports.

## Availability of data and meterials

All relevant datasets are available from the corresponding author upon reasonable request.

## Conflict of interest

None declared.

## Supplementary Material

Supplementary_1_tkae014
