# Supplementary material for: Pre-expanded muscle-sparing latissimus dorsi flap in defect reconstruction and its application strategy
Source: Burns Trauma. 2024 Jun 19;12:tkae014. doi: 10.1093/burnst/tkae014 (PMC11185896; doi:10.1093/burnst/tkae014)
Supplement: Supplementary_1_tkae014 [file supplementary_1_tkae014.docx]

Supplement 1.

Table 1.

|  | Age  (year) | Recipient site | Flap size  (cm x cm) | Expander volume  (ml) | Injection volume  (ml) | Expansion  duration  (month) | Type of MS-LD flap | Recipient vessel | Complications | Follow-up duration  (month) |
| --- | --- | --- | --- | --- | --- | --- | --- | --- | --- | --- |
| 1 | 29 | chest | 32x14 | 400 | 1880 | 13 | Type III | / | N | 18 |
| 2 | 22 | chest | 25x24 | 400 | 1050 | 6 | Type III | intercostal artery | N | 21 |
| 3 | 4 | chest | 25x15 | 400 | 1300 | 6 | Type I | / | N | 15 |
| 4 | 13 | chest | 30x15 | 400 | 1560 | 9 | Type I | / | N | 24 |
| 5 | 11 | shoulder | 25x17 | 400 | 1100 | 8 | Type I | / | N | 32 |
| 6 | 10 | chest | 20x11 | 400 | 1100 | 6 | Type I | / | N | 21 |
| 7 | 9 | neck | 27x17 | 400 | 1300 | 8 | Type II | facial artery | N | 12 |
| 8 | 27 | neck/shoulder | 30x21 | 500 | 1700 | 9 | Type I | / | N | 12 |
| 9 | 7 | shoulder/chest/axilla | 22x20 | 400 | 900 | 12 | Type I | / | N | 50 |
| 10 | 5 | chest | 23x13 | 300 | 1250 | 8 | Type I | / | expander ruprue | 42 |
| 11 | 21 | neck | 20x15 | 400 | 1300 | 9 | Type II | thyrocervical trunk | N | 23 |
| 12 | 5 | shoulder | 15x12 | 300 | 915 | 7 | Type I | / | N | 14 |
| 13 | 6 | arm | 35x20 | 600 | 2323 | 7 | Type II | contralateral TDA | Twisted fill tube | 35 |

Table1.

*N=NONE;
